# Multiplexing and massive parallel sequencing of targeted DNA methylation to predict chronological age

**DOI:** 10.3389/fragi.2025.1467639

**Published:** 2025-02-28

**Authors:** Bowen Zhu, Dean Li, Guojing Han, Xue Yao, Hongqin Gu, Tao Liu, Linghua Liu, Jie Dai, Isabella Zhaotong Liu, Yanlin Liang, Jian Zheng, Zheming Sun, He Lin, Nan Liu, Haidong Yu, Meifang Shi, Gaofang Shen, Zhaohui Hu, Lefeng Qu

**Affiliations:** ^1^ Interdisciplinary Research Center on Biology and Chemistry, Shanghai Institute of Organic Chemistry, Chinese Academy of Sciences, Shanghai, China; ^2^ University of Chinese Academy of Sciences, Beijing, China; ^3^ Department of Vascular and Endovascular Surgery, Chang Zheng Hospital, Naval Medical University, Shanghai, China; ^4^ Technology Department of Haidian Sub-Bureau, Beijing Public Security Bureau, Beijing, China; ^5^ Youyi Road Community Health Service Centre for Baoshan District, Shanghai, China; ^6^ Shanghai American School, Shanghai, China; ^7^ Forensic Science Institute of Shanghai Public Security Bureau, Shanghai, China; ^8^ Institute of Criminal Science and Technology Shanghai Xuhui Public Security Sub-Bureau, Shanghai, China; ^9^ Third Research Institute of Ministry of Public Security, Shanghai, China; ^10^ Institute of Criminal Science and Technology of Criminal Police Detachment, Yangzhou Public Security Bureau, Yangzhou, Jiangsu, China; ^11^ Department of Cardiovascular Diseases, Shanghai Punan Hospital of Pudong New District, Shanghai, China

**Keywords:** DNA methylation, age-prediction model, multiplexing, massive parallel sequencing, targeted region

## Abstract

Estimation of chronological age is particularly informative in forensic contexts. Assessment of DNA methylation status allows for the prediction of age, though the accuracy may vary across models. In this study, we started with a carefully designed discovery cohort with more elderly subjects than other age categories, to diminish the effect of epigenetic drifting. We applied multiplexing and massive parallel sequencing of targeted DNA methylation, which let us to construct a model comprising 25 CpG sites with substantially improved accuracy (MAE = 2.279, R = 0.920). This model is further validated by an independent cohort (MAE = 2.204, 82.7% success (±5 years)). Remarkably, in a multi-center test using trace blood samples from forensic caseworks, the correct predictions (±5 years) are 91.7%. The nature of our analytical pipeline can easily be scaled up with low cost. Taken together, we propose a new age-prediction model featuring accuracy, sensitivity, high-throughput, and low cost. This model can be readily applied in both classic and newly emergent forensic contexts that require age estimation.

## Introduction

Forensic DNA phenotyping exploits not just genetic polymorphisms but also epigenetic modifications, i.e., DNA methylation, to draw a bio sketch of an unknown subject (Kayser, 2015). DNA methylation is a methyl group on the cytosine (C) followed by guanine (G) that is commonly referred to as CpG site, where the p stands for the phosphodiester bond between the two nucleotides. Studies have reported models that estimated chronological age from DNA methylation status, i.e., the ratio between methylated and non-methylated CpG forms (Bekaert et al., 2015a; 2015b; Jung et al., 2019). These models profiled DNA methylation by low-throughput assays, i.e., pyrosequencing or PCR-based SNaPshot, thus limiting their ability to handle a large quantity of samples (Lee et al., 2015; Zbieć-Piekarska et al., 2015). Although models that used massive parallel sequencing have been developed, their prediction accuracy varied, usually deviating above 3 years from the actual age (Naue et al., 2017; Vidaki et al., 2017; Aliferi et al., 2018).

Mounting evidence has associated aging and diseases with loss of fidelity due to epigenetic drift, which involves the accumulation of changes in an individual’s epigenome over time (Salameh et al., 2020; Wu et al., 2021; Alimohammadi et al., 2022). The currently available age-prediction models involved the use of five to eight selected CpG sites, and consequentially their actual performance could be perturbed by aging and pathophysiological conditions (Zbieć-Piekarska et al., 2015; Freire-Aradas et al., 2016; Jung et al., 2019). Indeed, a substantial decline of prediction accuracy in the elder population has been documented in previous models (Zbieć-Piekarska et al., 2015). Notably, regardless of human ethical groups and applicable body fluids, these models are converged on CpG islands of ELOVL2, FHL2, KLF14, miR29B2C, and TRIM59 genes (Zbieć-Piekarska et al., 2015; Cho et al., 2017; Freire-Aradas et al., 2018; Jung et al., 2019; Han et al., 2022), suggesting that these regions may harbor DNA methylation with changes being mostly consistent with the progression of age, and that additional age-related CpG sites within the regions might be used to improve the accuracy of age prediction.

In the present study, we aim to increase the prediction accuracy by diminishing the effect of epigenetic drift. Hence, we devised a discovery cohort by deliberately recruiting more aged human subjects than other age categories. Our rationale lies at the fact that older individuals tend to have stochastic and sometimes conflicting changes of methylation status, such that only the most age-related markers throughout life course and neighboring CpG sites could be used for the age-prediction tool. It’s important to highlight that we assessed the performance of the model with an independent validation cohort and a multi-center test using trace blood samples taken directly from the forensic casework. We demonstrate that multiplexing of target regions, when combined with massive parallel DNA sequencing, enables the prediction of chronological age from blood samples with substantially improved precision, sensitivity, ease of bulk processing, and low cost.

## Material and methods

### The study population

This study was conducted in the Chinese Han ethnic group. We collected 318 blood samples, including 254 samples aged 30–70 years from the community of Shanghai Baoshan District, 55 samples aged 20–70 years from Shanghai Chang Zheng Hospital, 9 samples aged 50–70 years from Shanghai Pu Nan Hospital. These samples comprised 180 females and 138 males. Healthy individuals were recruited according to standardized procedures, such as physical health, mental wellbeing, and social adaptation, at baseline and follow-up visits from past 5 years. All blood samples were collected in Blood Nucleic Acids Tubes (Thermo Fisher, catalog: 4342792) and stored at −80°C until use, avoiding repeated freezing and thawing of plasma to prevent DNA degradation and contamination. Blood samples were used within 30 days from the time of collection. In addition, dried bloodstains from forensic casework were provided by the public security from Beijing (n = 5), Yangzhou (n = 59), and Shanghai (n = 8). For these samples, clinical records were not available. Dried bloodstains were used within half a year from the time of collection. Written informed consent was obtained prior to sample collection from every participant after explaining the objectives and procedures of the study.

### DNA extraction

For isolation of total DNA, 300 μL of whole blood was mixed with 3 μL RNase A (200 ng/μL, ABclonal, Catalog: RM29870) and 20 μL Proteinase K Solution (Magen, catalog: D6310-03B) and incubated for 15 min at 37°C with shaking. Bloodstains were cut and mixed with 20 μL Proteinase K Solution and 400 μL Digestive Solution ATL (Magen, catalog: D6310-03B) and then processed according to the manufacturer’s instructions (Magen, catalog: D6310-03B). DNA concentration was measured using NanoDrop (Thermo Fisher).

### Methylation analysis

Unmethylated cytosine was converted to uracil using the Bisulfite Conversion Kit (Singlera, catalog: EP110192). 200 ng- 1 μg of DNA was added with ddH_2_O to make up to 60 μL and then processed according to the manufacturer’s instructions (Singlera, catalog: EP110192).

Primers were designed by the website https://amplicondesign.dkfz.de/, with degenerate sequence as followings (R = A/G; Y=C/T): ELOVL2-F:5′- TACACGACGCTCTTCCGATCTYGGTYGGGYGGYGATTTGTA-3’; ELOVL2-R: 5′- GACGTGTGCTCTTCCGATCTACCCACCRAAACCCAACTAT-3’; miR29B2C-F: 5′- TACACGACGCTCTTCCGATCTGTAAATATATAYGTGGGGGAAGAAGGG-3’; miR29B2C-R: 5′- GAC​GTG​TGC​TCT​TCC​GAT​CTT​AAT​AAA​ACC​AAA​TTC​TAA​AAC​ATT​C-3’; TRIM59-F:5′- TACACGACGCTCTTCCGATCTTATYGGTGGTTTGGGGGAGAG-3’; TRIM59-R:5′- GACGTGTGCTCTTCCGATCTAACRACTTCCCRAAACAACRAATCTA-3’; KLF14-F:5′- TACACGACGCTCTTCCGATCTYGGTTTTYGGTTAAGTTATGTTTAATAGT-3’; KLF14-R:5′-GACGTGTGCTCTTCCGATCTCTACTACAACCCAAAAATTCC-3’; FHL2-F:5′- TACACGACGCTCTTCCGATCTTGTTTTTYGGGTTTTGGGAGTATAG-3’; FHL2-R:5′- GACGTGTGCTCTTCCGATCTCACRTCCTAAAACTTCTCCAATCTCC-3’.

The concentration of each primer was 100 μM. 4 μL each of ELOVL2-F/R, 3 μL each of miR29B2C-F/R, 2.9 μL each of TRIM59-F/R, 13.5 μL each of TRIM59-F/R, 8.5 μL each of FHL2-F/R and ddH_2_O 36.2 μL o were mixed with ddH_2_O to make up to 100 μL of primer mix. Library pre-construction was performed using KAPA2G Fast Multiplex Mix (Roche, catalog: 2GFMPXKB). PCR reactions were carried out in a total volume of 25 μL, containing 10 ng of converted DNA, 1 μL of primer mix and 12.5 μL of 2X KAPA2G Fast Multiplex Mix and ddH_2_O. The PCR program operated with an initial denaturation step of 5 min at 95°C, amplification for 25cycles (denaturation for 15 s at 95°C, annealing for 15 s at 58°C and extension for 30 s at 72°C), and a final extension for 5 min at 72°C. The amplified pre-hybridized libraries were then purified using VAHTS DNA Clean Beads (Vazyme, catalog: N411-02) with a volume ratio of 0.9 for the first round of sorting (DNA Clean Beads: DNA) and 0.3 for the second round of sorting. The purified products were subjected to secondary amplification using TaKaRa Ex Taq (TaKaRa, catalog: RR53A) and adaptor-specific primer (F: AAT​GAT​ACG​GCG​ACC​ACC​GAG​ATC​TAC​ACA​GCG​CTA​GAC​ACT​CTT​TCC​CTA​CAC​GAC​GCT​CTT​CCG​ATC​T; R: CAA​GCA​GAA​GAC​GGC​ATA​CGA​GAT​AAC​CGC​GGG​TGA​CTG​GAG​TTC​AGA​CGT​GTG​CTC​TTC​CGA​TCT). PCR reactions were carried out in a total volume of 25 μL, containing10 μl of purified products, 2.5 μL of 10×Ex Taq Buffer, 2 μL of dNTP, 1 μL of TaKaRa Ex Taq, 1 µL each of adaptor-specific primer F/R and 8.4 µL of ddH_2_O. The PCR program operated with an initial denaturation step of 5 min at 95°C, amplification for 13cycles (denaturation for 15 s at 95°C, annealing for 15 s at 58°C and extension for 30 s at 72°C), and a final extension for 5 min at 72°C. VAHTS DNA Clean Beads was used for purification with a volume ratio of 0.7 for the first round of sorting (DNA Clean Beads: DNA) and 0.3 for the second round of sorting. The concentration of the final library was determined using Qubit 2.0 (Invitrogen) Libraries were sequenced on the Illumina NovaSeq 6000 system (paired end; 150 bp).

### High-throughput sequencing data analysis

All sequencing reads were processed with Trim Galore (v0.6.6) (Krueger, 2015) with the parameters “--nextseq 30 --paired” to remove the adapter sequences (AGATCGGAAGAGC) from NovaSeq-platforms and reads longer than 20 bp were kept. Reads that passed the quality control procedure were kept and mapped to the *Homo sapiens* genome (GRCh38) using bismark (v0.24.1) (Krueger and Andrews, 2011) with default parameters. Uniquely mapped read pairs were extracted using samtools (v1.17) (Li et al., 2009) Methylation level was extracted by bismark.

### Age prediction model

To develop an age prediction model, we employed elastic net regression. Age prediction was trained by regressing chronological age on methylation level using the discovery cohort (N = 191). To begin, we randomly split the discovery cohort into training (70%) and test (30%) sets with balanced ages. Model optimization including hyperparameter tuning was done by a grid search with leave-one-out cross-validation (LOOCV) based on training sets. Model performance was assessed on the test set, using several statistics including median absolute error (MAE), Pearson’s correlation coefficient and its associated *p* value. Furthermore, we performed a cross-validation scheme for arriving the least biased estimates of the accuracy of the aging clocks, consisting of leaving out a single sample from the regression, predicting age for that sample, and iterating over all samples on the discovery cohort. The best-tuned hyperparameter α was 0.01, and λ was 1.2. Above model training and hyperparameter tuning were performed with R packages caret (v6.0–93) and glmnet (v4.1–4).

## Results

### The rational design of discovery aging cohort and multiplexing assay

**FIGURE 1:**
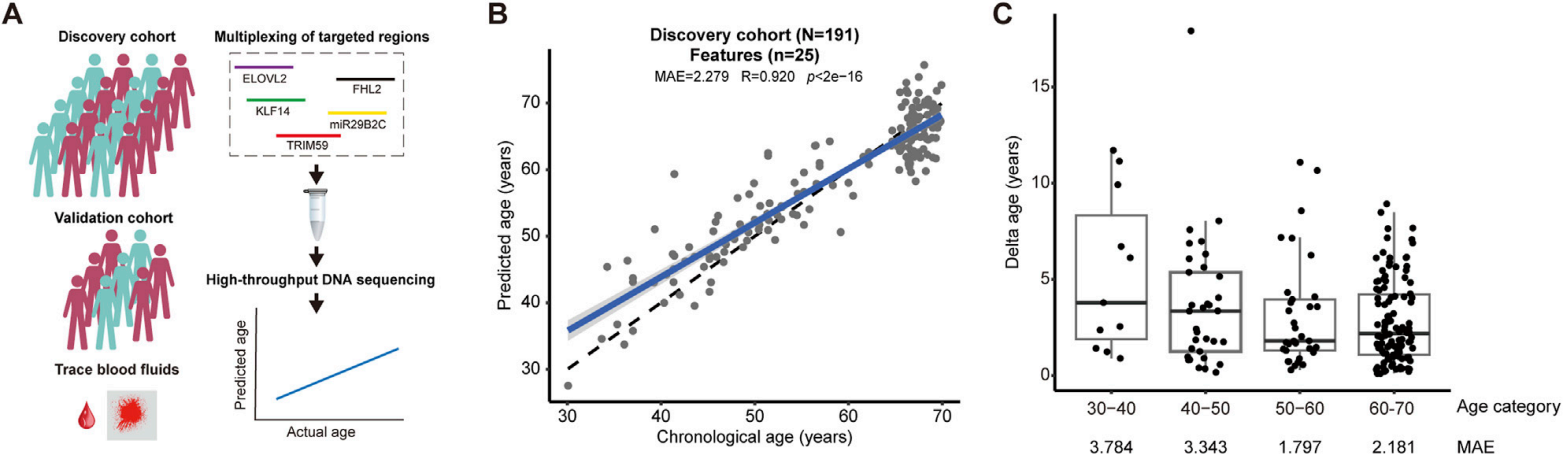
A new age-prediction model based on DNA methylation **(A)** We constructed an age-prediction model with a discovery cohort, which was consolidated by an independent validation cohort and a single-blind test using trace blood samples relevant to forensic casework (left panel). Our analytical pipeline was built on multiplexing of targeted DNA methylation from ELOVL2, FHL2, KLF14, miR29B2C, and TRIM59 gene, followed by high-throughput DNA methylation (right panel). **(B)** A new age-prediction model based on 25 CpG features was established using a discovery cohort consisting of 191 human subjects (MAE = 2.279, R = 0.920, *p* < 2e-16). **(C)** The accuracy of prediction shown as MAE in each age categories of the discovery cohort.

To establish a discovery cohort, we collected 191 peripheral blood samples from volunteers of Chinese Han ethnicity at the Baoshan District community of Shanghai. We deliberately recruited more aged subjects equal to or older than 60 years compared to other age categories (Figure 1A; Supplementary Figure S1A). Our rationale lies at the fact that elderly individuals, due to medical history and age, may couple with epigenetic drifting that confounds DNA methylation status, such that only CpG sites that are mostly correlated with age could be selected. Different from previous reports that relied on low-throughput assay, we introduced multiplexing PCR reaction followed by massive parallel DNA sequencing, thereby allowing the assessment of all CpG sites from the select regions of ELOVL2, FHL2, KLF14, miR29B2C, and TRIM59 genes (Figure 1A). We consolidated the feasibility of the analytical pipeline. First, we performed PCR reaction to confirm the specificity of primer sets, as evidenced by single PCR products for each target region (Supplementary Figure S1B). Second, we subjected the product of multiplexed PCR reaction for high-throughput DNA sequencing. Sequence analysis demonstrated sufficient read counts, with minimally more than 1,000 for each region involved (Supplementary Figure S1C). Third, we assessed DNA methylation status for all target regions, revealing CpG sites that displayed discordant changes with age. Taken together, this data establishes that age-related CpG sites from target regions could be used to develop an aging prediction model.

### A new age-prediction model

We constructed a new age-prediction model using the discovery cohort and a pipeline based on multiplexing of target regions and massive parallel DNA sequencing (Figure 1A). First, we assessed the ability of age-prediction by using reported CpG sites by Zbieć-Piekarska et al. (2015); Jung et al. (2019), respectively, and found that the use of limited CpG sites led to models with median absolute error (MAE) higher than 3 years and variance accountant for age (R) lower than 0.89 (Supplementary Figure S2). Second, we applied elastic net regression and cross-validation to construct a new age-prediction model that involved 25 CpG sites, including 2 from miR29B2C, 8 from FHL2, 5 from TRIM59, 9 from ELOVL2, and 1 from KLF14, respectively, with 18 new CpG features not being used by previous models (Table 1). This model demonstrated significantly improved accuracy with MAE as low as 2.279 (R = 0.920, *p* < 2e-16) (Figures 1B, C).

**TABLE 1:** A new age-prediction model based on 25 CpG sites A list of CpG sites used in the age-prediction model, including 2 from miR29B2C, 8 from FHL2, 5 from TRIM59, 9 from ELOVL2, and 1 from KLF14, respectively. CpG sites used by previous models were highlighted in bold blue.

Locus	Gene function	CpG site	Chromosome location (GRCh38)	Regression coefficient
miR29B2C	non-coding RNA	**C1**	**chr1:207823681**	−0.031
		C2	chr1:207823705	−0.0242
FHL2	Transcription factor	**C1**	**chr2:105399282**	0.0354
		**C2**	**chr2:105399288**	0.1146
		C3	chr2:105399297	0.0561
		C4	chr2:105399300	0.039
		C5	chr2:105399327	−0.0837
		C6	chr2:105399360	−0.6492
		C7	chr2:105399363	−0.2134
		C8	chr2:105399388	0.057
TRIM59	Regulator of immune signaling pathways	C1	chr3:160450179	0.1184
		C2	chr3:160450184	0.0528
		**C3**	**chr3:160450189**	0.138
		C4	chr3:160450192	0.0748
		**C5**	**chr3:160450199**	0.0824
ELOVL2	Synthesis of very long chain polyunsaturated fatty acids	**C1**	**chr6:11044628**	0.1533
		C2	chr6:11044634	0.2957
		C3	chr6:11044640	0.0894
		C4	chr6:11044644	0.0852
		C5	chr6:11044647	0.1189
		C6	chr6:11044655	0.0631
		**C7**	**chr6:11044661**	0.2512
		C8	chr6:11044683	−0.0028
		C9	chr6:11044702	−0.0712
KLF14	Transcription factor	C1	chr7:130734398	0.2441

### Validation by an independent cohort

**FIGURE 2:**
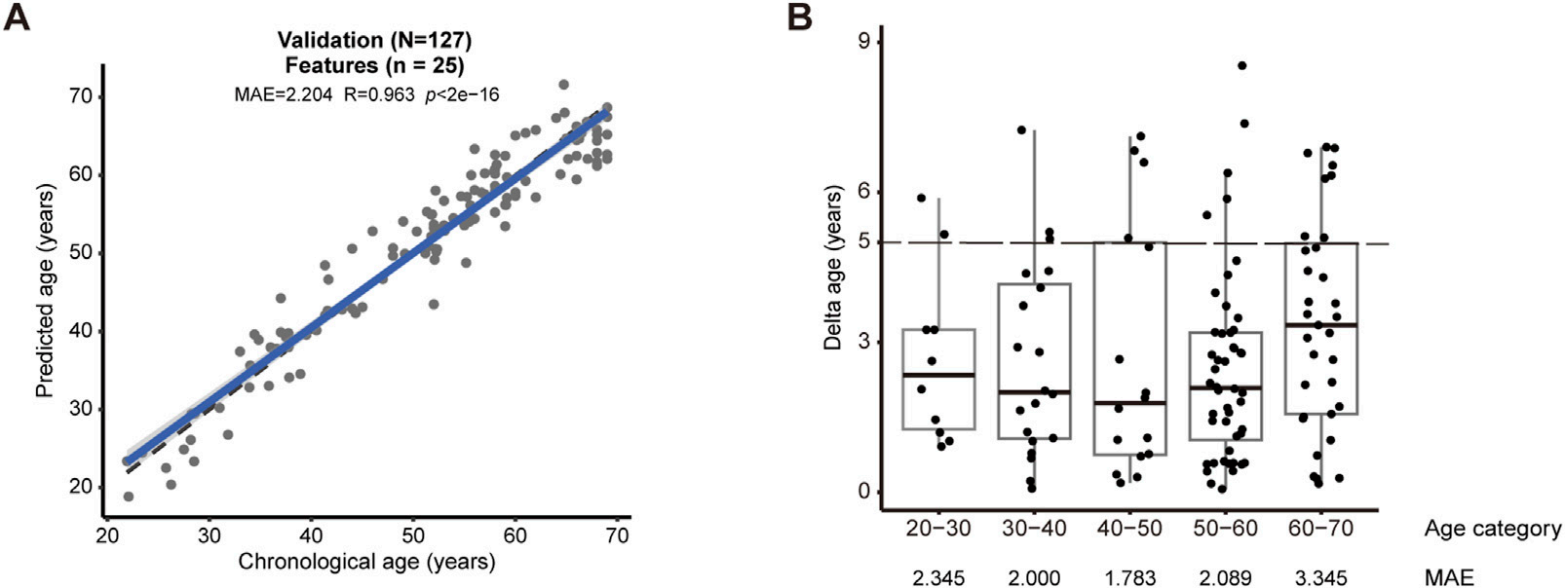
Validation by an independent cohort **(A)** The validation cohort consisting of 127 human subjects confirmed the accuracy of the model (MAE = 2.204, R = 0.963, *p* < 2e-16). **(B)** The accuracy of prediction in each age category of the validation cohort. Successful prediction could reach 82.7%, when the deviation between predicted versus actual age was set as 5 years (dashed lines). MAE was shown for each age categories.

We validated the new model in an independent validation cohort comprising 127 subjects. This experiment showed that our model could reach 2.204 for MAE (R = 0.963, *p* < 2e-16) (Figure 2A). When the maximum difference between predicted and actual age was set as 5 years, we observed 82.7% successful predictions for the entire cohort (Figure 2B), and notably 89.6% success for the age category between 50 and 60 (Figure 2B).

### Efficacy to predict age from trace blood samples

**FIGURE 3:**
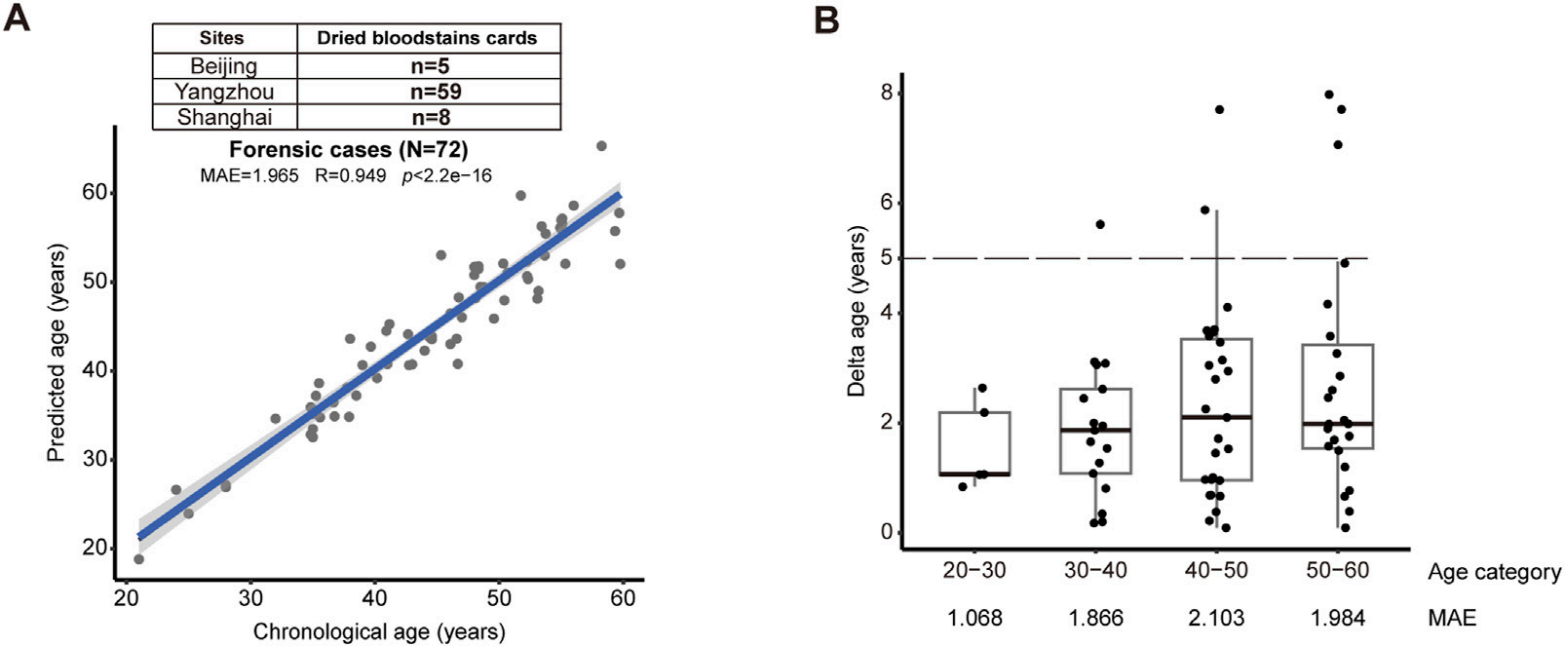
Validation by trace blood samples from forensic casework **(A)** The validation cohort consisting of 72 dried bloodstains subjects confirmed the accuracy of the model (MAE = 1.965, R = 0.949, *p* < 2.2e-16). **(B)** Successful prediction could reach 91.6% from bloodstains and trace blood fluids, when the deviation between predicted versus actual age was set as 5 years (dashed lines). MAE was shown for each age categories.

We attested the model with trace blood samples that were practically relevant to the crime scenes in the real world. We initiated a multi-center test using dried bloodstains from forensic casework provided by public security from Beijing (n = 5), Yangzhou (n = 59), and Shanghai (n = 8) (Figure 3A). Without prior knowledge of age, our model could reach 1.965 for MAE (R = 0.949, *p* < 2.2e-16) (Figure 3A). Notably, when the maximum difference between the predicted and actual age was set as 5 years, our data demonstrated 91.7% success (Figure 3B). Comparatively, our new model has improved accuracy than previous age-prediction tools also based on massive parallel sequencing technique (Supplementary Table S1). Significantly, this result illustrates the ability of our model, together with the streamlined analytical pipeline, to handle trace blood samples, strongly supporting its readiness in forensic application.

## Discussion

Estimation of age is informative in many forensic contexts, e.g., classically used in investigative leads for crime scenes (Phillips, 2015). The demand of this application is now rapidly growing in age categorization of illegal immigrants as well as asylum seekers in which context valid identification documents are usually missing (Schmeling et al., 2016). Thus far, forensically practical age-prediction models have been established by the assessment of DNA methylation status, known as epigenetic clock (Hannum et al., 2013; Horvath, 2013; Florath et al., 2014). The currently available models involve the use of 5-8 CpG sites and low-throughput analytical platforms, e.g., pyrosequencing (Weidner et al., 2014; Bekaert et al., 2015b; 2015a; Huang et al., 2015). In the present study, we apply multiplexing of target regions followed by high-throughput DNA sequencing. Trained by a carefully designed aging cohort, we propose a new epigenetic clock model that includes 25 age-related CpG sites.

Epigenetic DNA methylation is one of the hallmarks of aging, laying critical foundation for its application to estimate the chronological age of human subjects. However, it is well-known that stochastic changes in DNA methylation occur with age, smoking habit, alcoholic consumption, and disease situations (Johansson et al., 2013; Hagerty et al., 2016; Spólnicka et al., 2018b; 2018a; Yang et al., 2019). Consequently, an age-related increase in interindividual variability and reciprocally a decline in accuracy have been reported in many age-prediction models based on biomarkers of DNA methylation (Bekaert et al., 2015a; 2015b; Zbieć-Piekarska et al., 2015; Park et al., 2016). Hence, we designed a training cohort by deliberately recruiting more elderly subjects than other age categories, such that our model could be built with CpG sites that are mostly consistent with the progression of age. Presumably, the accuracy of age prediction could be substantially improved by increasing the number of DNA methylation loci. As such, we focus on 5 genomic regions with CpG sites therein repeatedly used by various early models (Zbieć-Piekarska et al., 2015; Freire-Aradas et al., 2018; Jung et al., 2019; Woźniak et al., 2021; Aliferi et al., 2022; Han et al., 2022). By high-throughput DNA sequencing, we can obtain profiles of all DNA methylation loci from which we construct a new model comprising 25 CpG sites. Despite increased number of DNA methylation loci, our pipeline has been simplified by multiplexing of these 5 target regions. Moreover, the nature of our analytical pipeline, by measuring the ratios between methylated and non-methylated CpG from high-throughput sequencing, with minimally 1000 read counts for each region, supports the consistency and robustness of the result. Empowered by this new model, we could predict age with a success rate of 82.7% (±5 years) in an independent validation cohort and a success rate of 91.7% (±5 years) in a multi-center test using dried bloodstains taken from real world forensic casework.

Compared to the early models based on low-throughput assay, we apply high-throughput sequencing, together with multiplexing of target regions, thus allowing bulk processing of large volume of samples. This ability is of paramount urgency, given the exponentially increasing casework of illegal immigrants and asylum seekers resulted from geographic conflict. Moreover, another issue to consider is cost. It is important to note that the cost for massive parallel DNA sequencing has been significantly reduced, especially feasible for batch testing. Taken together, we propose a new age-prediction model featuring substantially improved accuracy, sensitivity, ease of bulk processing, and low cost.

## Conclusion

In this study, we propose a new age-prediction model, when combined with multiplexing of targeted DNA methylation and massive parallel sequencing, that has substantially improved accuracy, ability to handle trace blood samples, ease of large-scale application, and low cost. To conclude, this model can be readily applied in both classic and newly emergent forensic contexts that require the estimation of chronological age.

## Data Availability

The datasets presented in this study can be found in online repositories. The names of the repository/repositories and accession number(s) can be found below: https://www.ncbi.nlm.nih.gov/geo/, GSE267985.
