# Supplementary material for: Multiplexing and massive parallel sequencing of targeted DNA methylation to predict chronological age
Source: Front Aging. 2025 Feb 28;6:1467639. doi: 10.3389/fragi.2025.1467639 (PMC11906720; doi:10.3389/fragi.2025.1467639)
Supplement: Supplementary file 1 [file DataSheet1.docx]

**
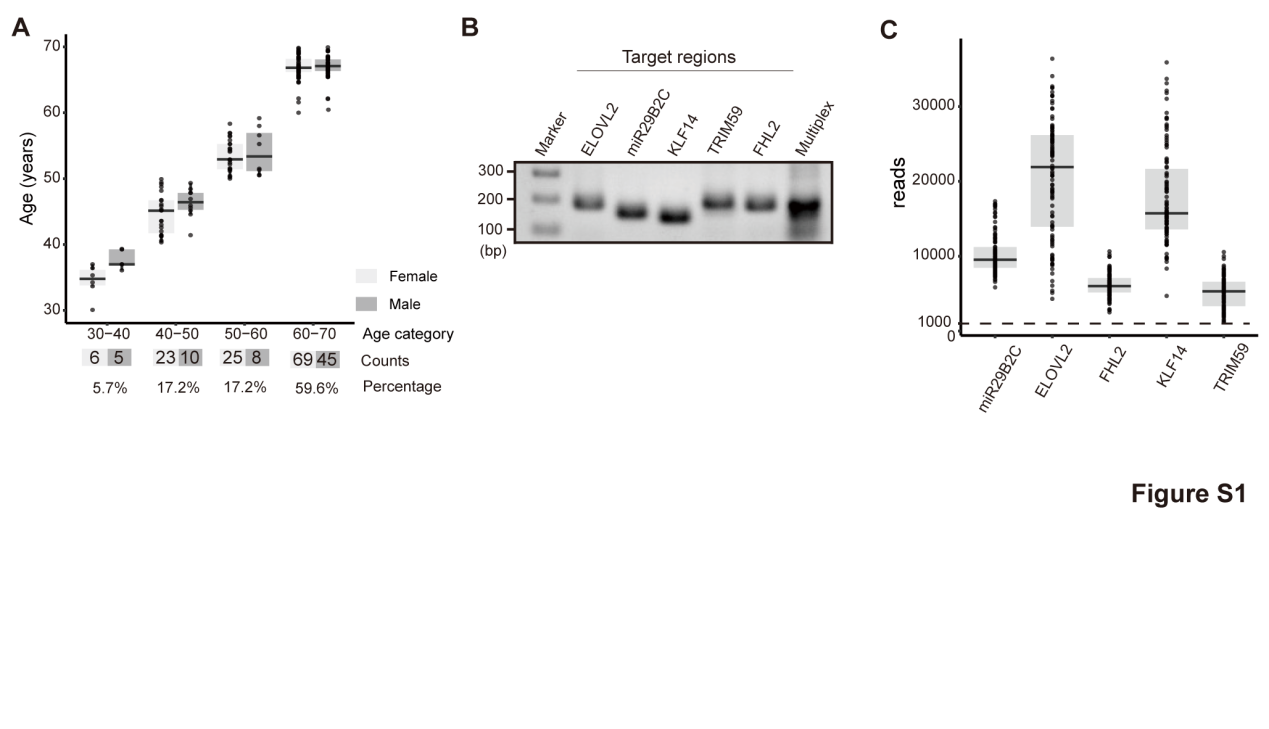
**

**Supplementary Figure 1.** (A) Age distribution of the discovery cohort, with more elderly subjects than other age categories. (B) Individual versus multiplexing of PCR reaction of target regions from miR29B2C, FHL2, TRIM59, ELOVL2, and KLF14 genes. (C) High-throughput sequencing of multiplexing of target regions, with minimally 1,000 read counts for each region (dashed line), supports the consistency and robustness of the result.

**
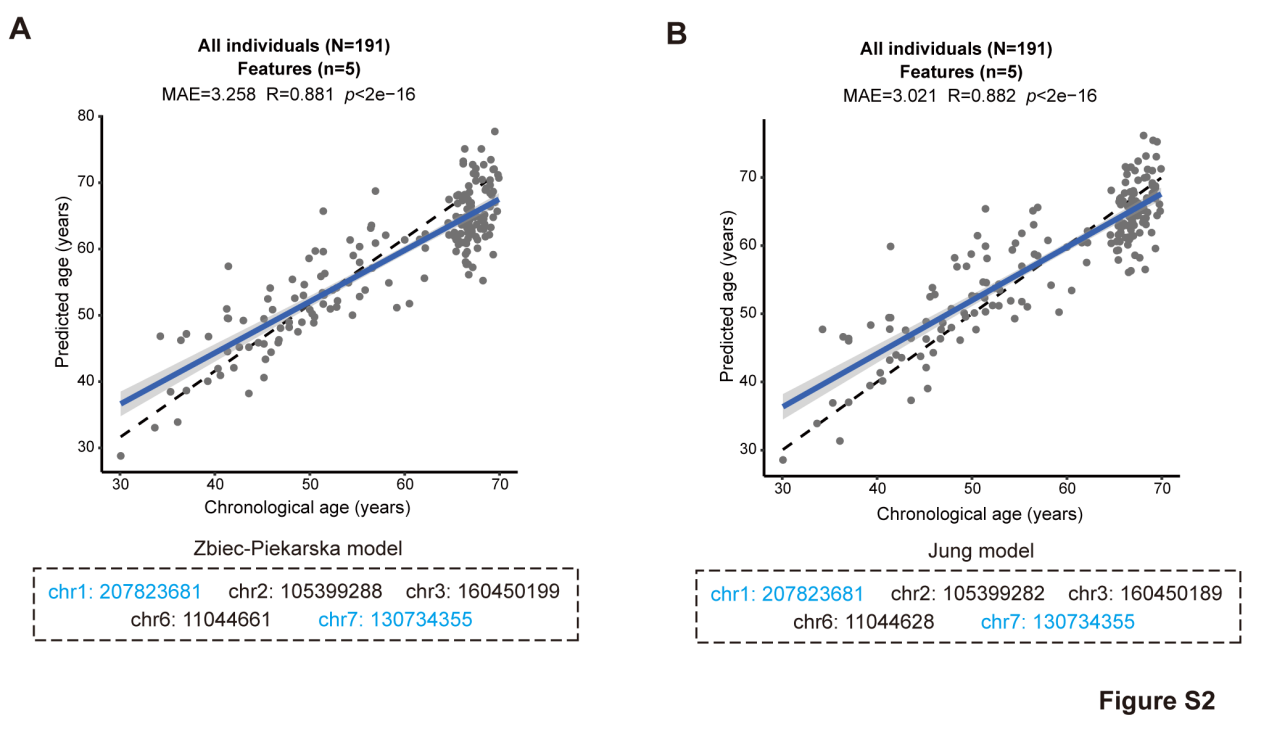
**

**Supplementary Figure 2.** Age-prediction models using CpG sites previously published by Zbiec-Piekarska et al. (A) and Jung et al. (B). CpG sites used in both models were highlighted in blue.

**Supplementary Table 1.** Performance comparison between the current model and previous age-prediction models based on massive parallel sequencing.

| **Source** | **Group** | **Number of samples** | **MAE (years)** |
| --- | --- | --- | --- |
| Current study | Discovery cohort | 191 | 2.279 |
|  | Validation cohort | 127 | 2.204 |
|  | Trace blood samples | 72 | 1.965 |
| Aliferi et al. | Training set | 76 | 4 |
|  | Blind test set | 33 | 4.1 |
| Naue et al. | Training set | 208 | 3.21 (MAD) |
|  | Test set | 104 | 3.16 (MAD) |
| Vidaki et al. | Training set | 1,156 | 3.3 |
|  | Test set | 231 | 4.4 |
